# Exploring the untapped pharmacological potential of imidazopyridazines

**DOI:** 10.1039/d3ra07280k

**Published:** 2024-01-29

**Authors:** M. Shaheer Malik, Hossa F. Alshareef, Khalid A. Alfaidi, Hissana Ather, Zainularifeen Abduljaleel, Essam M. Hussein, Ziad Moussa, Saleh A. Ahmed

**Affiliations:** a Department of Chemistry, Faculty of Science, Umm Al-Qura University Makkah 21955 Saudi Arabia; b Science and Technology Unit, Umm Al-Qura University Makkah 21955 Saudi Arabia; c Department of Pharmaceutical Chemistry, College of Pharmacy, King Khalid University (KKU) Abha 62529 Saudi Arabia; d Department of Chemistry, Faculty of Science, Assiut University 71516 Assiut Egypt; e Department of Chemistry, College of Science, United Arab Emirates University P.O. Box 15551 Al Ain United Arab Emirates msmalik@uqu.edu.sa saahmed@uqu.edu.sa

## Abstract

Imidazopyridazines are fused heterocycles, like purines, with a pyridazine ring replacing the pyrimidine ring in purines. Imidazopyridazines have been primarily studied for their kinase inhibition activity in the development of new anticancer and antimalarial agents. In addition to this, they have also been investigated for their anticonvulsant, antiallergic, antihistamine, antiviral, and antitubercular properties. Herein, we review the background and development of different imidazopyridazines as potential pharmacological agents. Moreover, the scope of this relatively less charted heterocyclic scaffold is also highlighted.

## Abstract 2

Imidazopyridazines are fused heterocycles, like purines, with a pyridazine ring replacing the pyrimidine ring in purines.

## Graphical abstract

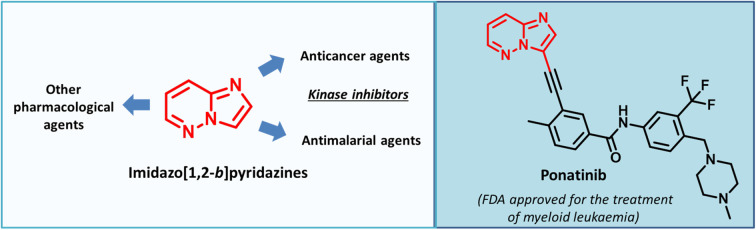

## 1. Introduction

**Fig. 1:**
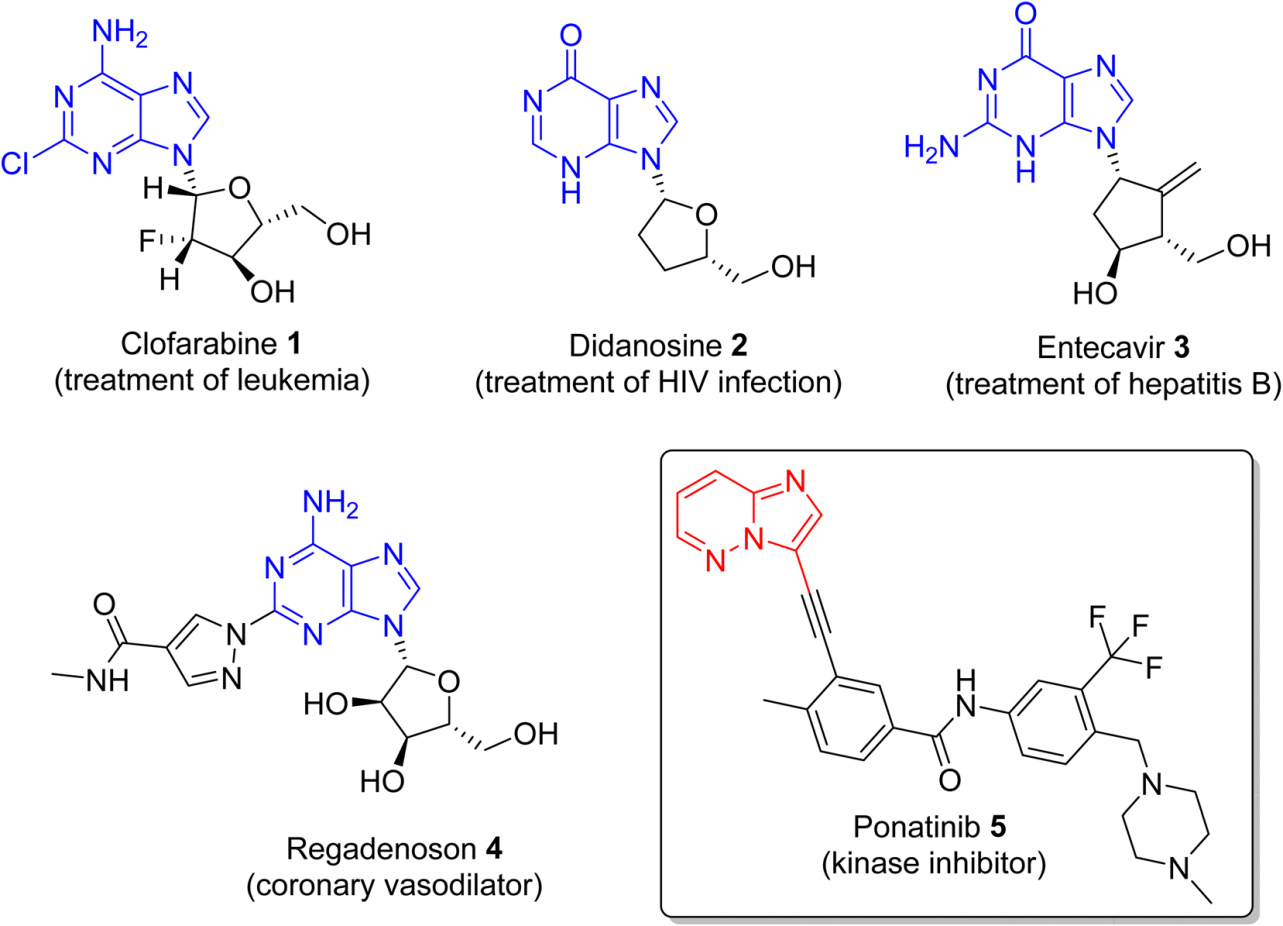
Drugs with imidazopyrimidine (purine) and imidazopyridazine rings (see inset).

Nitrogen-containing heterocyclic compounds form the backbone of the pharmaceutical industry. Reports show that nearly three quarters of the drugs approved by the Food and Drug Administration (FDA) contain nitrogen-based heterocyclic compounds with a plethora of pharmacological applications that include anti-infectives, anti-metabolites, stimulants, depressants and replenishing agents.^1–3^ The robust application of nitrogen-based heterocyclic compounds stems from the fact that the nitrogen atom can readily accept or donate protons and also participate in diverse non-bonding interactions, which include hydrogen bond formation, and dipole–dipole, hydrophobic, van der Waals, and π-stacking interactions.^4^ These interactions can result in the high binding affinity of nitrogen-based compounds to different enzymes and biological receptors. In addition, in nature, nitrogen-based heterocycles are key constituents in diverse vital molecules like proteins, enzymes, vitamins, and nucleic acids. The essential nitrogenous bases (guanine, cytosine, adenine, and thymine) of DNA and RNA contain nitrogen-containing heterocyclic systems, namely purines and pyrimidines. Different nitrogen-containing heterocyclic scaffolds are known to be biologically active, which includes unfused, fused and bridged scaffolds.^1^ One of the important fused heterocycles is purine in which an imidazole and a pyrimidine ring are fused together. It is present naturally in nucleic acids (DNA and RNA) and in synthetic drugs such as clofarabine 1 (treatment of leukaemia), didanosine 2 (treatment of AIDS), entecavir 3 (hepatitis B virus inhibitor), regadenoson 4 (coronary vasodilator), *etc.*, (Fig. 1).^5–8^ The imidazopyridazine heterocycle is a counterpart of the purine-based imidazopyrimidine moiety that differs in the location of the two nitrogen atoms in the six-membered ring. It is not a part of any vital molecules in nature and the pharmacological potential of the imidazopyridazine framework has not been extensively investigated. The research endeavour into the potential applications of this scaffold started with the FDA approval in 2012 of the imidazopyridazine derivative ponatinib (5) as an anticancer drug.^9^ Ponatinib (5) is a tyrosine kinase inhibitor indicated for use in treating chronic myeloid leukemia.^10^

**Fig. 2:**
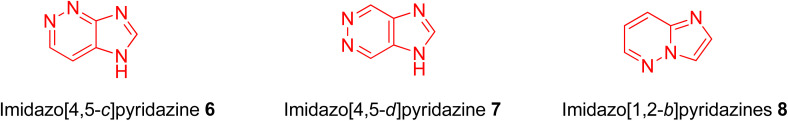
Different imidazopyridazine rings.

**Fig. 3:**
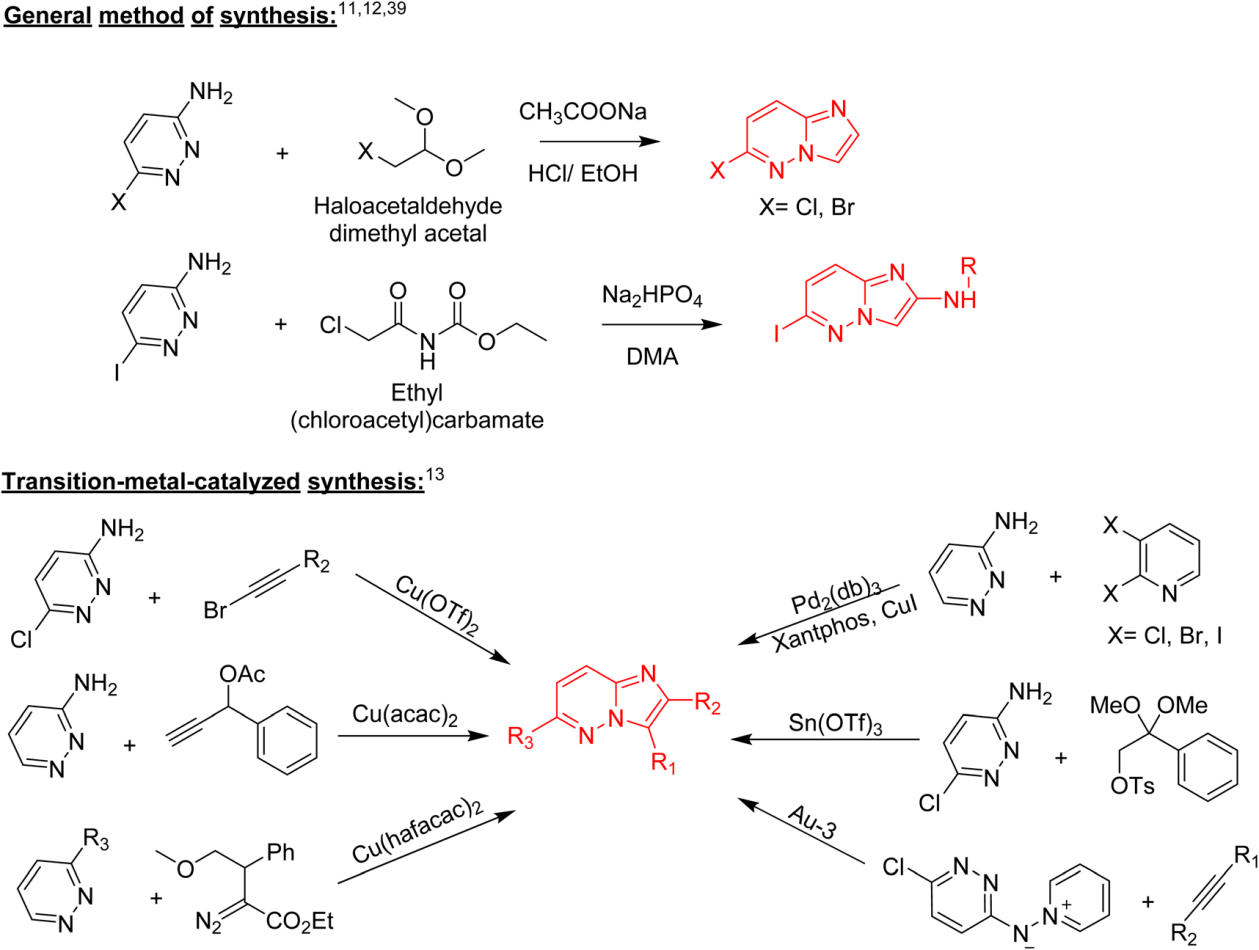
Some synthetic routes to imidazo[1,2-*b*]pyridazines.

Structurally, imidazopyridazine is a fused heterocycle that contains an imidazole and a pyridazine ring. The fusion of these heterocyclic rings can result in different isomers namely imidazo[4,5-*c*]pyridazine (6) and imidazo[4,5-*d*]pyridazine (7) both of which has two nitrogen atoms in each ring. In addition, a third possible structure is imidazo[1,2-*b*] pyridazines (8), in which the one nitrogen atom is shared by both the imidazole and pyridazine rings (Fig. 2). The imidazo[1,2-*b*]pyridazines have been comparatively more deeply explored than imidazo[4,5-*c*]pyridazines and imidazo[4,5-*d*]pyridazines. Therefore, the synthetic strategies to imidazo[1,2-*b*]pyridazines are more developed compared to other counterparts. Some of the general methods included the condensation of appropriately substituted pyridazine rings with haloacetaldehyde dimethyl acetal or ethyl (chloroacetyl)carbamate to provide imidazo[1,2-*b*]pyridazine derivatives (Fig. 3).^11,12^ In recent years, transition-metal catalysed synthetic routes to access imidazo[1,2-*b*]pyridazine derivatives from appropriate starting materials are increasingly being employed with established cross-coupling reactions.^13^ In this copper catalysed reaction are extensively developed using catalyst with acetylacetonate, hexafluoroacetylacetonate and triflate ligands. This is followed by palladium-based metal catalyst and some other metal catalyst based on tin and gold are also reported.

As mentioned imidazo[1,2-*b*]pyridazines are widely explored compared to imidazo[4,5-*c*]pyridazines and imidazo[4,5-*d*]pyridazines. The pharmacological potential of these imidazo[1,2-*b*]pyridazines has been investigated by different research groups;^14,15^ however, the developments in all the imidazopyridazine isomers have not been reviewed. In the present review, we focus on the development of imidazopyridazine derivatives as potential pharmacological agents, which will be helpful to readers interested in exploring this relatively less charted area by designing of the new imidazopyridazine-based compounds.

Imidazopyridazines have been reported to exhibit mostly anticancer and antimalarial properties, but additional pharmacological properties have also been investigated. For clarity, we have categorized this review into imidazopyridizines as anticancer, antimalarial, and other pharmacological agents.

## 2. Imidazopyridazines as anticancer agents

**Fig. 4:**
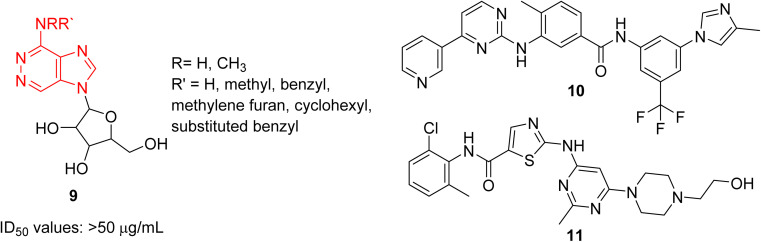
Imidazopyridazine 9 as anticancer agents and subsequent development.

Cancer remains as one of the most deadly human diseases, characterized by a notably high occurrence and fatality rate.^16^ In recent years, the emergence of drug resistance towards the clinically used anticancer drugs has further aggravated the situation.^17^ Therefore, a constant need of new anticancer drug discovery and development programs is a prerequisite to address the challenges posed by cancer. Bussolari and co-workers were the first to explore the anticancer properties of imidazopyridazine derivatives by designing the series of *N*^4^-substituted imidazo[4,5-*d*]pyridazine nucleosides 9 along with *v*-triazolo[4,5-*d*]-pyridazine counterparts (Fig. 4).^18^ Different substituents, including methyl, benzyl, methylene furan, cyclohexyl and substituted benzyl, were incorporated on the amino group on the *N*-4 position. The nucleosides were evaluated for cytotoxicity against murine L1210 leukemia and B16 melanoma cells. The results showed that the imidazo[4,5-*d*]pyridazine nucleosides displayed ID_50_ values of >50 μg mL^−1^ against the tested cancer cell lines which compared to ID_50_ values of 0.1 μg mL^−1^ for the standard drugs. For a considerable period, there was a conspicuous lack of reports on imidazopyridazines as anticancer agents. This changed in 2012 as FDA approved a new multi-targeted, tyrosine kinase inhibitor, ponatinib (5), which possesses an imidazo[1,2-*b*]pyridazine moiety, for the treatment of chronic myeloid leukemia (CML).^9^ Ponatinib (5) is a third generation kinase inhibitor that overcomes the T3151 mutation and has potent inhibitory effect against BCR-ABL1 kinases and numerous ABL1 mutations. It is primarily indicated for CML that is resistant to other kinase inhibitors like nilotinib 10 and dasatinib 11.^19,20^

**Fig. 5:**
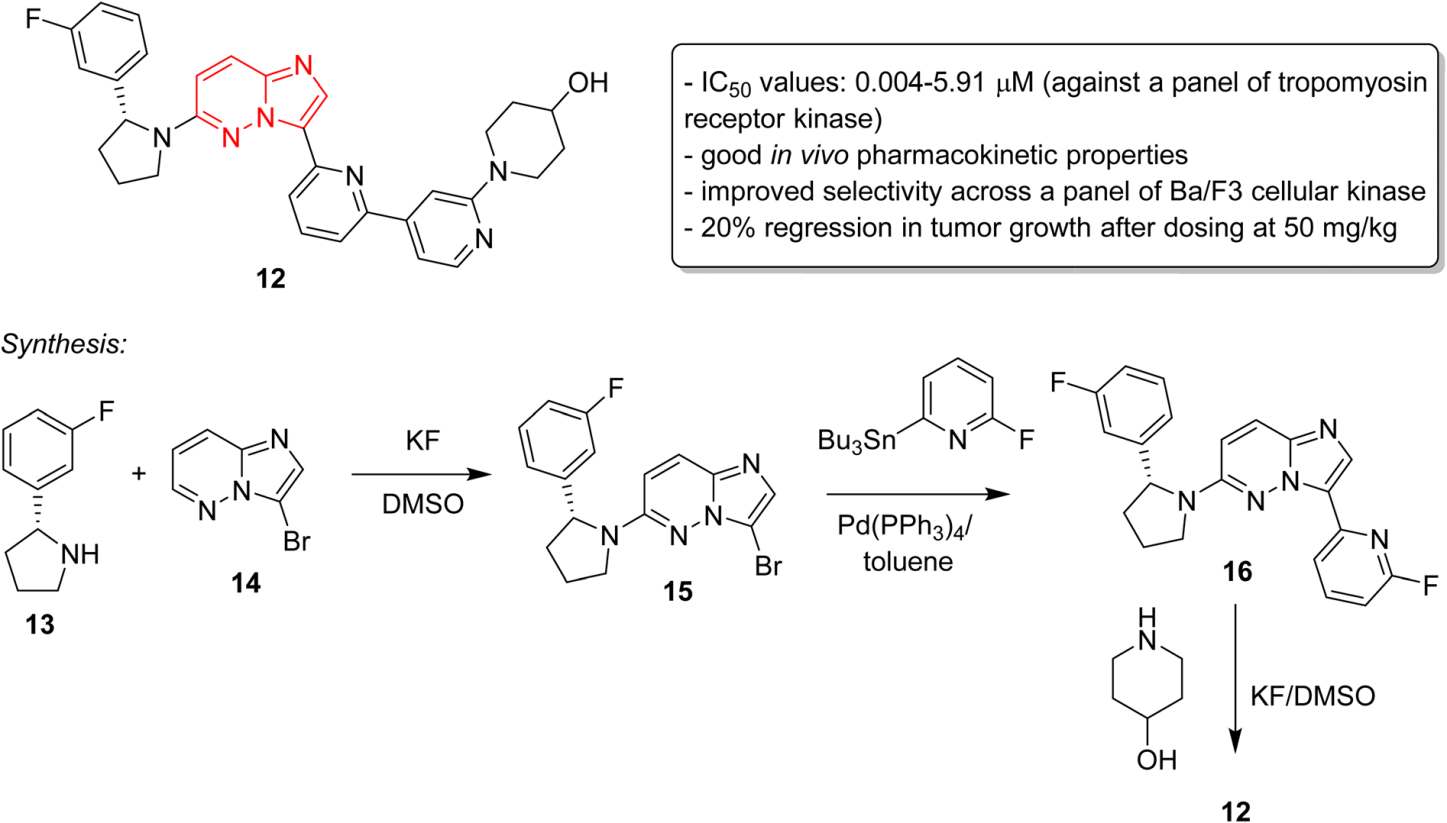
Imidazopyridazines as selective pan-TRK inhibitors: synthesis of active inhibitor 12.

The discovery of ponatinib sparked an imperative momentum towards exploring imidazopyridazines as potent kinase inhibitors in the development of new anticancer agents. Nerve growth and blood supply is the cornerstone of the microenvironment of a tumor. Cancer cells release neurotrophins, a nerve growth factor (NGF), by perturbing the neurotrophin signaling.^21^ Tropomyosin receptor kinase receptors (TRKA, TRKB, and TRKC) as well as their respective neurotrophin ligands are essential elements of neurotrophin signaling and the inhibition of TRKs is a sound strategy in the development of anticancer agents.^22,23^ Choi and co-workers, a research group at Novartis, screened an in-house compound collection and identified a novel imidazopyridazine as an inhibitor of tropomyosin receptor kinase.^24^ Structure-guided drug design revealed (*R*)-2-phenylpyrrolidine substituted imidazopyridazines as a novel class of selective pan-TRK inhibitors. The best molecule was GNF-8625 (12) which displayed IC_50_ values of 0.004–5.91 μM on testing against a panel of kinases, TRKA, TRKB, TRKC and WT (Fig. 5). It showed good *in vivo* pharmacokinetic properties in a rat model, and improved selectivity across a panel of Ba/F3 cellular kinase, along with few other molecules. It also induced a 20% regression in tumor growth after dosing at 50 mg kg^−1^ and partially inhibited tumor growth in a dose-dependent manner. Chemistry optimization was carried out to identify new chemical compounds with good *in vivo* profile and over fifteen compounds were synthesized by applying different chemical strategies. The most potent compound 12 was synthesized by a three step procedure involving the reaction between 3-bromo-6-chloroimidazo[1,2-*b*]pyridazine 13 and (*R*)-2-(3-fluorophenyl)pyrrolidine 14 to afford intermediate 15 that was subjected to Stille reaction resulting in compound 16. Finally, the condensation of 16 with piperidin-4-ol provided the active imidazopyridazine 12.

**Fig. 6:**
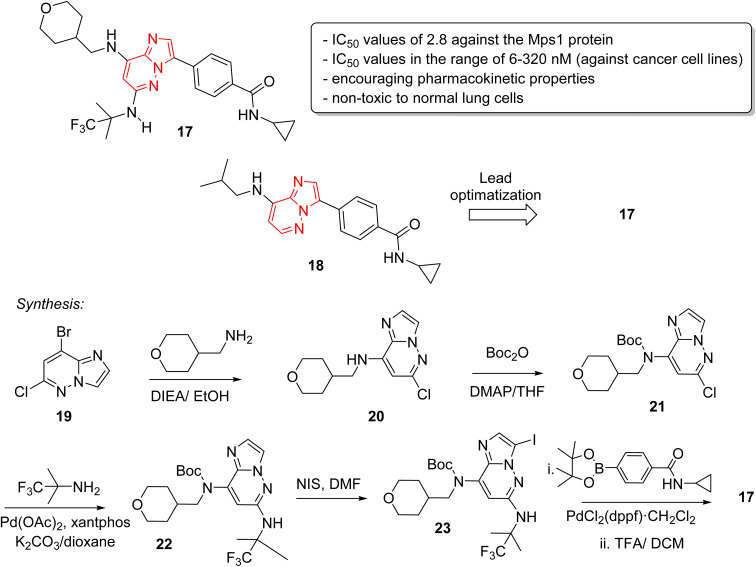
Imidazopyridazines as Mps1 (TTK) kinase inhibitors: synthesis of active inhibitor 17.

Monopolar spindle 1 (Mps1) or TTK, is a kinase that phosphorylates different amino acid residues and is a vital constituent of the spindle assembly checkpoint, thereby of importance in mitotic cell division. The levels of Mps1 and TTK are elevated in human malignancies and it has emerged as a novel and druggable target in cancer.^25^ Kusakabe and co-workers identified and synthesized a selective Mps1 inhibitor 17 with outstanding anticancer potency starting from a high throughput screening hit containing an imidazo[1,2-*a*]pyrazine ring (Fig. 6).^26^ The structure-based design of molecules from the HTS hit produced several other Mps1 inhibitors along with compound 17. The research group carried out a high throughput screening and identified an imidazo[1,2-*a*]pyrazine 18 that showed good biochemical activity but other potency such as cellular and antiproliferative needed improvement. Lead optimization was carried out by co-crystallization technique which guided the introduction of substituents at the 6-position for enhancement of activity. A series of stepwise optimization studies was carried out and the best result was shown by compound 17. The imidazo[1,2-*a*]pyrazine 17 exhibited excellent IC_50_ values of 2.8 and 6.0 nM against the Mps1 protein and A549 cancer cell line, respectively. It also showed encouraging pharmacokinetic properties in Sprague-Dawley rats. Antiproliferation assay of compound 17 against a panel of 14 cancer cell lines from four different cancers showed excellent results with IC_50_ values in the range of 6–320 nM. A notable aspect was that it was non-toxic toward normal lung cells. The synthetic approach to the 17 is outlined in Fig. 5 starting with bromo imidazopyridazine derivative 19 that was condensed with tetrahydro-2*H*-pyran-4-yl)methylamine to provide 20. Compound 20 was boc-protected to give 21 and a palladium acetate catalyzed condensation provided 22. Iodination at position 3 of scaffold resulted in 23, which was converted to the potent inhibitor 17 in two subsequent steps.

**Fig. 7:**
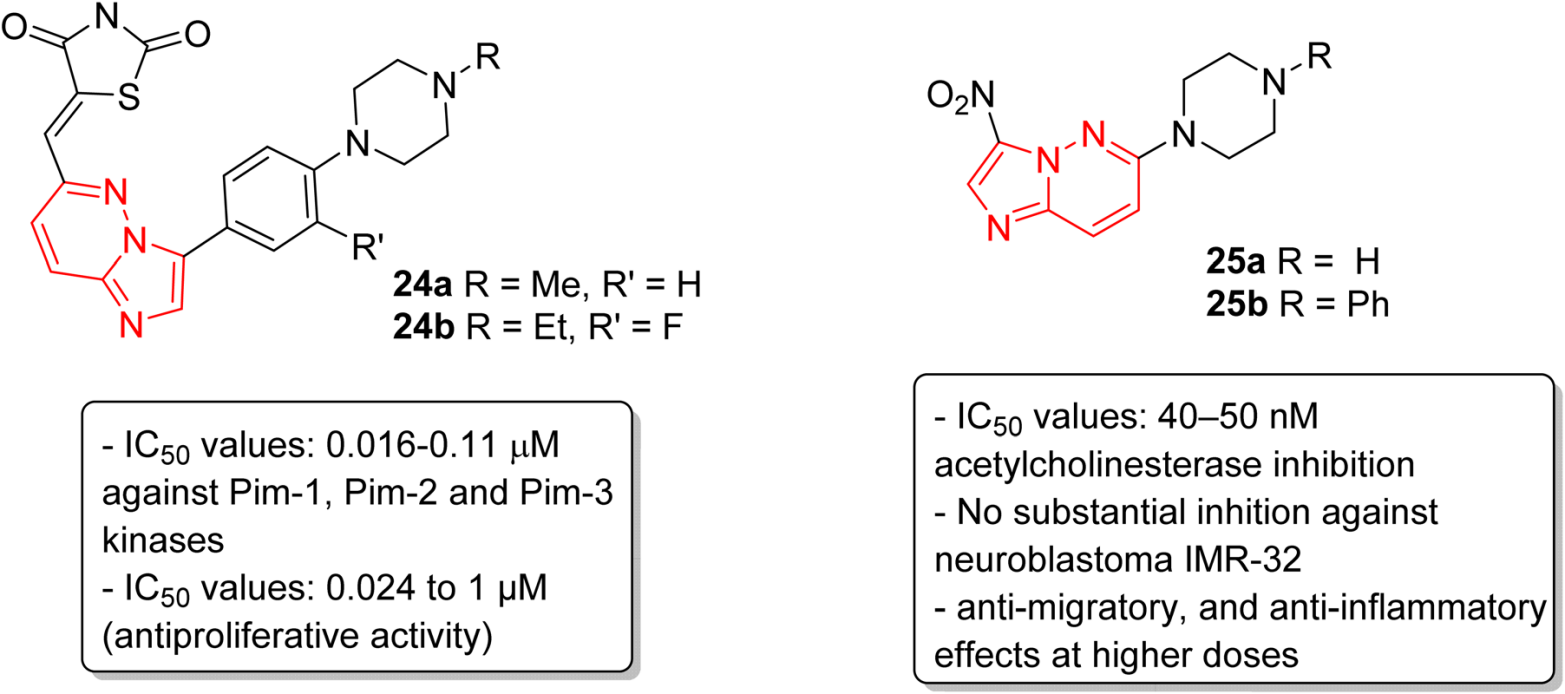
Imidazopyridazines as Pim kinases and acetylcholinesterase inhibitors.

Pim kinases, a class of serine/threonine kinases, are essential in various cell-signaling pathways and are reported to be overexpressed in different types of cancers.^27^ Sawaguchi and coworkers examined the preclinical characteristics of a series of imidazopyridazine thiazolidinedione conjugates.^28^ Two of the conjugates 24a and 24b exhibited the best IC_50_ values which were in the range of 0.016–0.11 μM against Pim-1, Pim-2 and Pim-3 kinases (Fig. 7). Conjugates 24a and 24b also exhibited a broad spectrum anti-proliferative activity with IC_50_ values of 0.027 to 1.0 μM and 0.024 to 0.65 μM, respectively. Additionally, they suppressed phosphorylation of Pim kinase substrates, caused arrest of G1 phase cell cycle, and induced apoptosis. Moreover, they displayed antitumor activities in xenograft models. In another report, Sharma and co-workers described the synthesis by incorporating nitro and amino functionalities designed to enhance the range of the biological activity of imidazopyridazines.^29^ Two of the imidazopyridazines, (25a and 25b) showed potent inhibition of acetylcholinesterase activity (IC_50_ = 40–50 nM) and inhibited proliferation of the neuroblastoma IMR-32cell line, with 25b being more potent, and enhanced caspase 3 activity. These compounds also arrested cells in G0/G1 phase, decreased ATP levels, and increased mitochondrial oxidative stress. Additionally, both imidazopyridazines showed anti-inflammatory activity as the result of significant inhibition of LPS-induced COX-2 and iNOS expression.

**Fig. 8:**
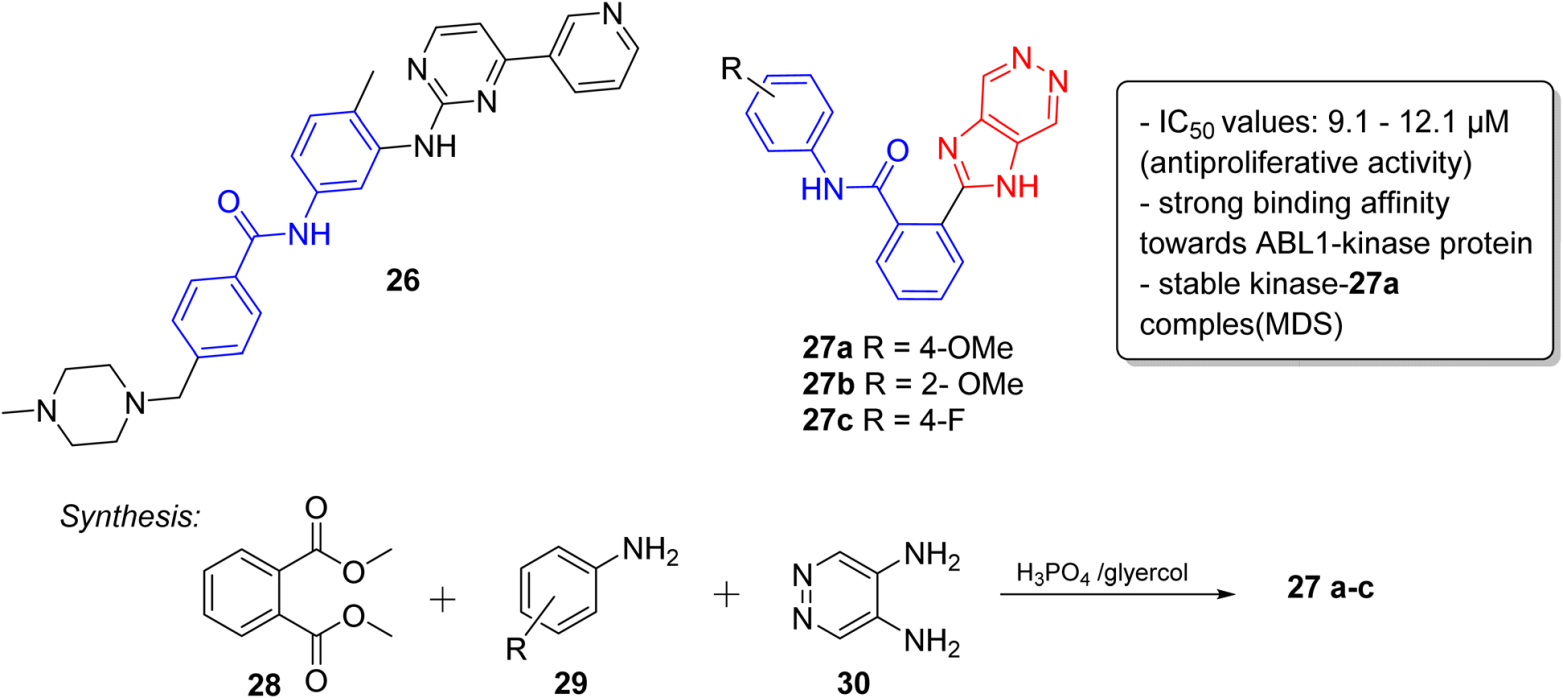
Imidazopyridazines as antiproliferative agent.

Lately, the tyrosine kinase inhibitors developed as new anticancer drugs, including imatinib (26), nilotinib (10), and ponatinib (5), are based on the striking feature of an *N*-phenylbenzamide core with a nitrogen-containing heterocyclic system.^30^ Taking this into consideration, very recently, we have designed and synthesized a series of novel imidazopyridazine-based *N*-phenylbenzamide derivatives as potential anticancer agents.^31^ The cytotoxic evaluation of these derivatives against selected cancer cell lines demonstrated that the imidazopyridizine 27a was most potent from the series, and it displayed IC_50_ values that were lower than 9.1 μM (Fig. 8). The imidazopyrues in the range of 10.2 to 12.1 μM. Docking experiments suggested that the imidazopyridizine 27a exhibited a strong binding affinity towards ABL1-kinase protein like the control compound nilotinib and molecular dynamic simulations indicated that the protein-27a complex was as stable as the control. The imidazopyridazine-based *N*-phenylbenzamide derivatives were synthesized by employing a one-pot multicomponent approach involving dimethyl phthalate 28, aniline 29 and pyridazine-4,5-diamine 30 to obtain active products 27a–c. The multicomponent reaction was catalyzed by phosphoric acid with glycerol as solvent.

## 3. Imidazopyridazines as antimalarial agents

**Fig. 9:**
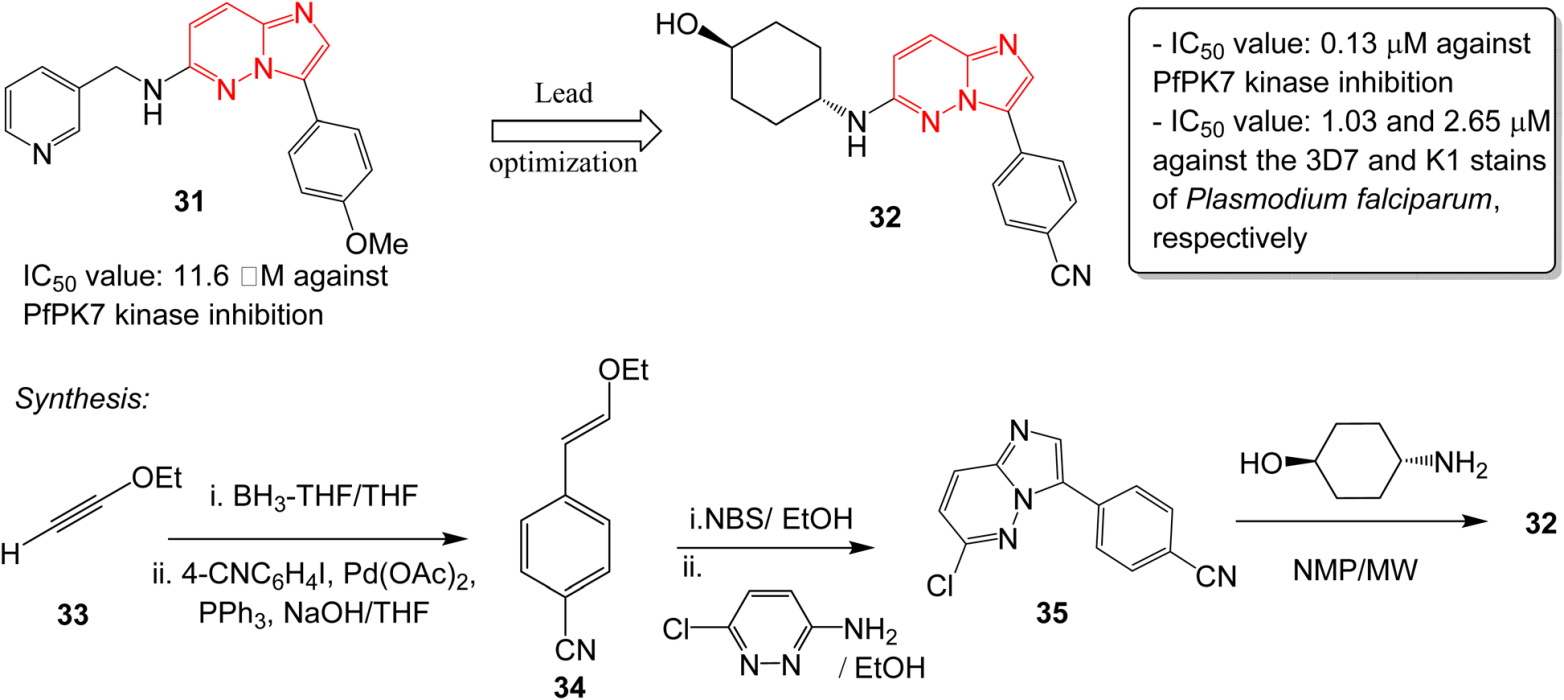
Imidazopyridazines as PfPK7 kinase inhibitor and antimalarial agent. Synthesis of active compound 32.

Malaria is a disease caused by the parasite, *Plasmodium falciparum*, and is a one of the foremost public health issues in tropical and subtropical regions. Malarial infections are not typically fatal (<1%); however, the infection is responsible for half a million deaths annually.^32^ The high rate of incidence and emergence of resistance highlights the need for developing new antimalarial drugs. Heterocyclic compounds form the crux of antimalarial drug discovery programs.^33^ A plasmodial kinase, PfPK7, is an emerging target in antimalarial drug design that is related to the MAPKK family of kinases.^34^ Bouloc and co-workers identified several imidazopyridazines as novel PfPK7 kinase inhibitors through a high-throughput screening campaign.^35^ Previously, a research group identified a hit imidazopyridazine 31 as a weak inhibitor of PfPK7 kinase with an IC_50_ value of 11.6 μM.^36^ The optimization of the hit 31 was carried out by Bouloc and co-workers and this led to the identification of the potent PfPK7 inhibitor 32 that displayed an IC_50_ value of 0.13 μM. It also exhibited IC_50_ values of 1.03 and 2.65 μM against the 3D7 and K1 stains of *Plasmodium falciparum*, respectively (Fig. 9). The synthesis started with one-pot hydroboration and cross-coupling of alkyne 33 with 4-iodobenzonitrile to afford 34. This was followed by bromination and condensation with pyridazine derivative providing intermediate 35. Finally, condensation with the appropriate chiral amine resulted in the potent compound 32. Considering the importance of PfPK7 kinase inhibitors, Sahu and co-workers rationally designed a series of imidazopyridazine derivatives using 2D and 3D structural models to provide structural insights into the design more potent PfPK7 kinase inhibitors.^37^

**Fig. 10:**
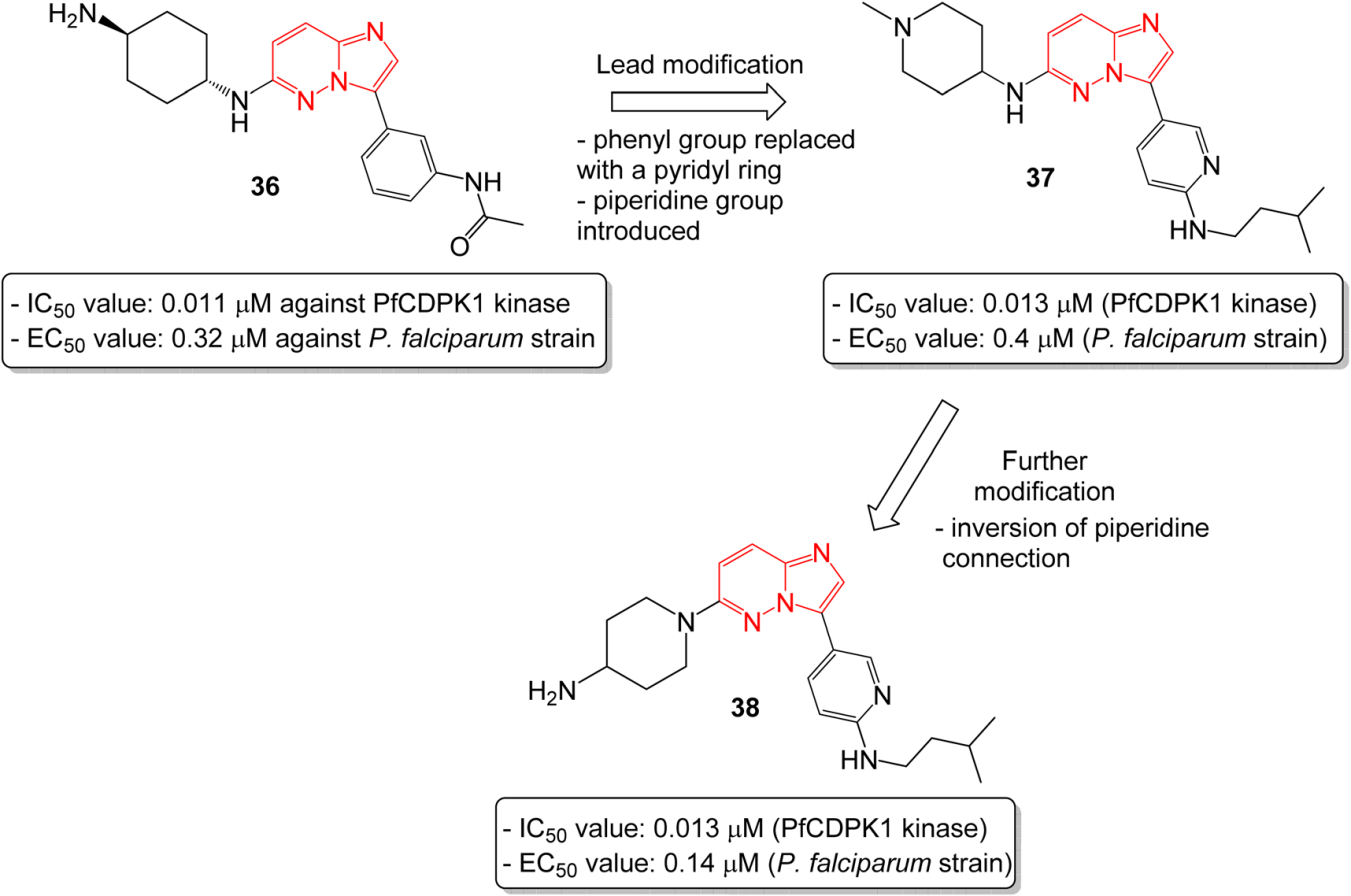
Imidazopyridazines as PfCDPK7 kinase inhibitor and antiplasmodial agent.

In another report, Chapman and co-workers identified a series of imidazopyridazines as effective inhibitors of *Plasmodium falciparum* calcium-dependent protein kinase 1 (PfCDPK1) by a high throughput screening method.^38^ Initial screening led to the identification of imidazopyridazine 36 with *N*-linked phenyl amide and carbamate groups that showed an IC_50_ value of 0.011 μM against PfCDPK1 and an EC_50_ value of 0.32 μM against a *P. falciparum* strain (Fig. 10). To further improve the physicochemical properties of the compounds, the phenyl group attached to imidazopyridazine ring was replaced with a pyridyl ring and a piperidine ring was introduced in place of cyclohexyl ring. This afforded good enzyme affinity, high cell potency and a satisfactory ADME profile. The best compound was imidazopyridazine 37 that displayed an IC_50_ value of 0.013 μM against PfCDPK1 and an EC_50_ value of 0.4 μM against a *P. falciparum* strain. Further modification of 37, by inversion of the piperidine linkage to the ring, resulted in imidazopyridazine 38 with identical PfCDPK1 inhibition (IC_50_ = 0.013 μM) and the best EC_50_ value of 0.14 μM against the *P. falciparum* strain. Overall, the modification resulted in a two-fold increase in potency against *P. falciparum* strain.

**Fig. 11:**
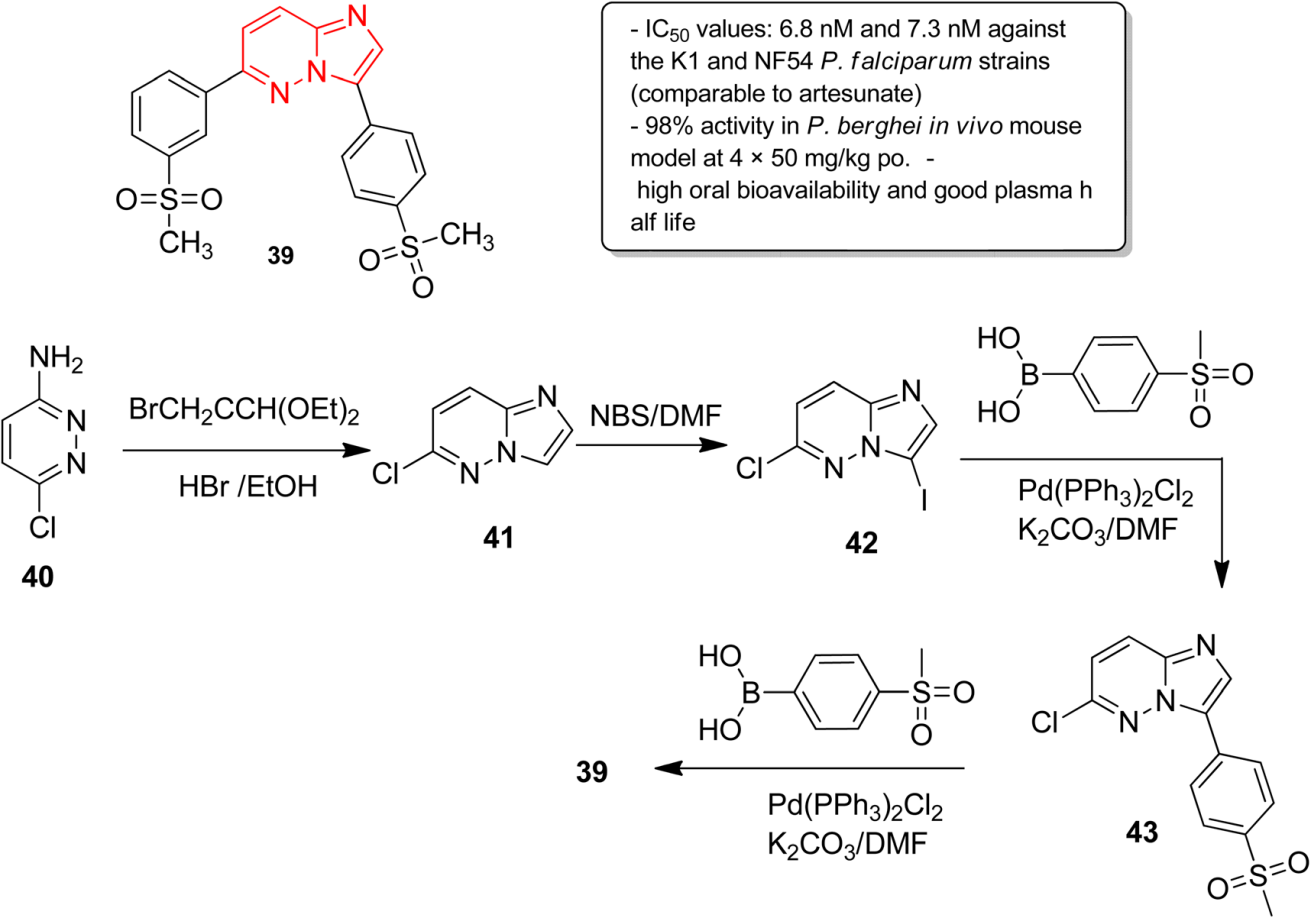
Imidazopyridazines as antiplasmodial agent. Synthesis of active compound 39.

In another report, Manach and co-workers screened a SoftFocus kinase library and identified three imidazopyridazine hits active at nanomolar concentration.^39^ Further evaluation of *in vitro* ADME properties showed poor metabolic stability of the imidazopyridazine hits. Taking two of the imidazopyridazine hits as the starting point, extensive structure activity relationship studies were conducted. Several novel imidazopyridazines were synthesized and tested for antiplasmodial activity against a multidrug resistant (K1) and drug sensitive (NF54) *P. falciparum* strain. These studies resulted in the identification of several highly active molecules of which the most potent was imidazopyridazine derivative 39 (Fig. 11). It exhibited IC_50_ values of 6.8 nM and 7.3 nM against the K1 and NF54 *P. falciparum* strains, respectively, and the activity was comparable to the known antimalarial drug, artesunate. It also displayed 98% activity in *P. berghei* mouse (*in vivo* model) and high oral bioavailability along with good ADME properties. The imidazopyridazine derivative 39, with excellent potency against both drug-sensitive and drug-resistant strains of *P. falciparum*, serves as a novel antimalarial drug lead molecule. The synthesis of the active compound 39 started with imidazopyridine ring formation involving 3-amino-6-chloropyridazine 40 and bromoacetaldehyde diethylacetal to provide the fused ring 41. Iodination of the 41 provided the intermediate 42, which was subjected to Suzuki cross coupling with appropriate boronic acid to afford 43. Finally, the second Suzuki cross coupling with appropriate boronic acid resulted in the active compound 39.

**Fig. 12:**
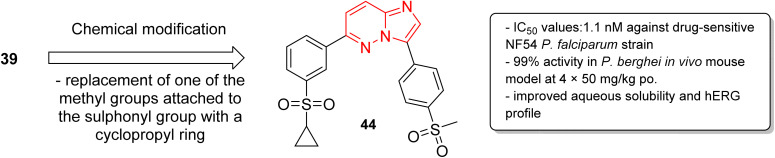
Development of potent antiplasmodial imidazopyridazine 44.

In a second part, the same research group carried out further optimization by the synthesis of over forty imidazopyridazine derivatives to identify molecules with enhanced aqueous solubility, improved hERG profile, and enhanced metabolic stability.^40^ The *in vitro* and *in vivo* studies demonstrated that replacement of one of the methyl groups attached to the sulphonyl group in 39 with a cyclopropyl ring resulted in enhanced potency. The imidazopyridazine derivative 44 displayed a remarkable IC_50_ value of 1.1 nM against the drug sensitive NF54 plasmodium strain (Fig. 12). It displayed 99% activity in *in vivo P. berghei* mouse model and cured all the three malaria-infected mice. Moreover, it considerably enhanced aqueous solubility, metabolic stability, and the hERG inhibitory profile compared to the earlier lead molecule 39.

A major limitation of 39 is the low aqueous solubility and to address this Noonan and co-workers reported various new supramolecular products, inclusive of a number of multicomponent solid forms.^41^ Three cocrystal forms with dicarboxylic acid coformers were prepared and characterized by thermal methods and single-crystal X-ray diffraction. A tailored solubility experiment was performed, and all of the multicomponent forms demonstrated an enhanced maximum concentration attained by the drug lead and the rate of dissolution.

## 4. Imidazopyridazines as other pharmacological agents

**Fig. 13:**
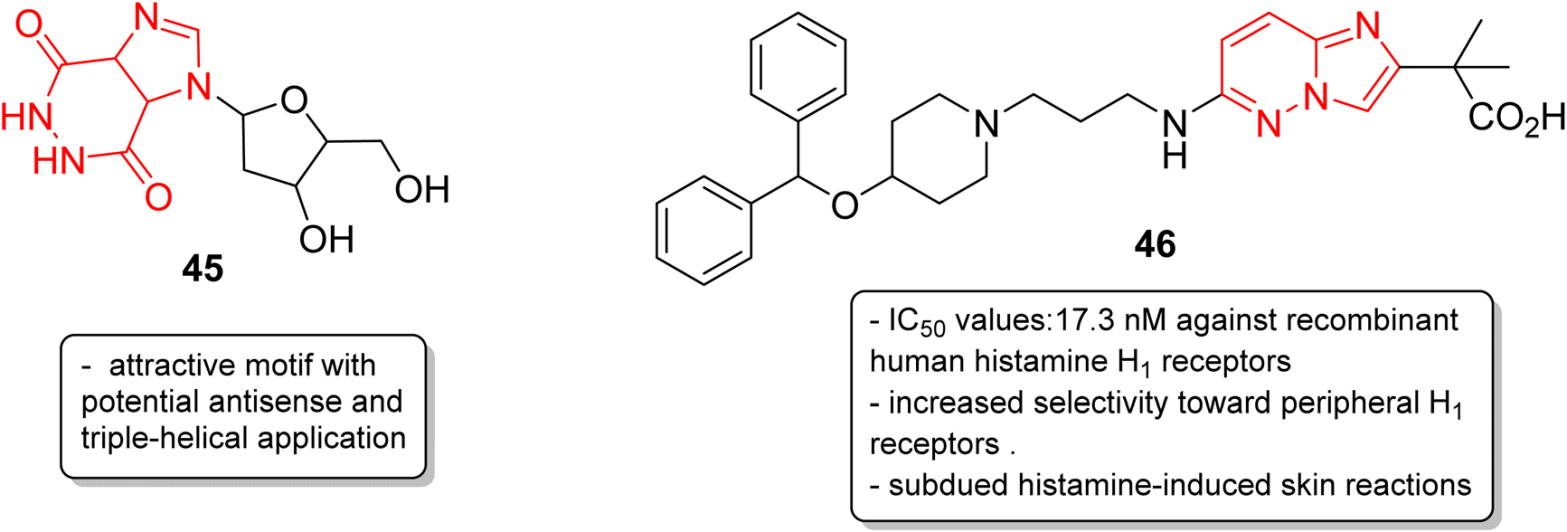
Imidazopyridazines as antisense and antihistamine agent.

In addition to anticancer and antimalarial properties, imidazopyridazine derivatives have also been reported to express other pharmacological activities, including anticonvulsant, antiallergic, antihistamine, antiviral, antitubercular and anti-psoriasis effects. Kelly and co-workers were the first to investigate the anticonvulsant activity of imidazopyridazine derivatives. They synthesized imidazo[4,5-*c*]pyridazines as well as imidazo[4,5-*d*]pyridazines and evaluated their anticonvulsant activity against maximal electroshock-induced seizures, with limited potential.^42^ Chen and co-workers described a synthetic procedure for the ribofuranosyl derivative of imidazo[4,5-*d*]pyridazine 45 and the molecular docking studies suggested that it could be an attractive motif with potential antisense and triple-helical application (Fig. 13).^43^ Antihistamines are extensively used to treat allergic symptoms developed by the release of biogenic histamines in the body.^44^ Fukuda and co-workers carried out an extensive screening study and discovered an imidazopyridazine derivative 46 that suppressed inflammation in guinea pigs.^45^ In the following year, the same research group reported the antihistamine effect of the new imidazopyridazine derivative 46 to treat allergic disorders.^46,47^ The *in vitro* binding profile of 46 against recombinant human histamine H_1_ receptors (rhH_1_R) demonstrated that it inhibited ligand binding to rhH_1_R with an IC_50_ value of 17.3 nM. Compound 46 displayed increased selectivity toward peripheral H_1_ receptors similar to that of the control and also subdued histamine-induced skin reactions in guinea pigs and mice. Moreover, additional studies suggested it could have long-lasting antihistamine potency with fewer side effects such as sedation and allergic reactions.

**Fig. 14:**
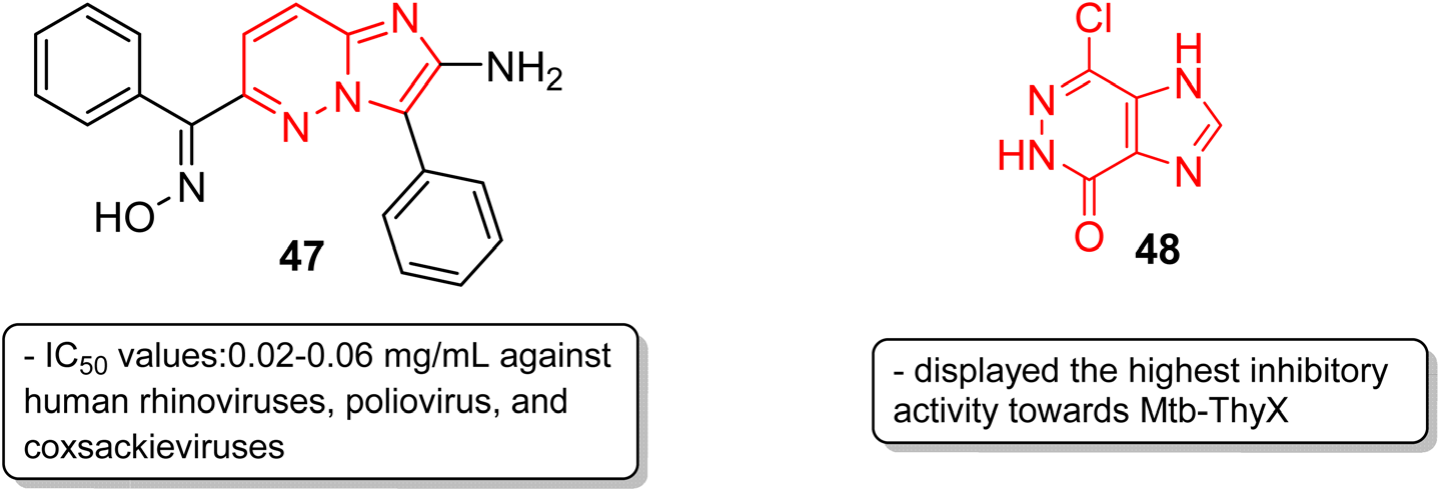
Imidazopyridazines as antiviral and anti-tubercular agents.

Picornavirus infections result in several acute and chronic diseases including poliomyelitis, hepatitis, myocarditis and others.^47^ Hamdouchi and co-workers reported a class of picornavirus inhibitors with an imidazo[1,2-*b*]pyridazine scaffold.^48^ The synthesis and antiviral evaluation of a series of 2-aminoimidazo[1,2-*b*]pyridazines revealed that 47, with an oxime functionality, was the most active and promising molecule (Fig. 14). It displayed a broad-spectrum activity against a panel of viruses comprising human rhinoviruses, poliovirus, and coxsackieviruses. The IC_50_ values were in the range of 0.02–0.06 mg mL^−1^. Tuberculosis is a dreadful mycobacterial infection and a high degree of drug-resistant tuberculosis strains have emerged.^49^ Flavin-dependent thymidylate synthase (ThyX) is an important transferase enzyme and the *Mycobacterium tuberculosis* contains genes to encode it, thereby making it a druggable target.^50^ Luciani and co-workers carried out virtual screening of Mtb-ThyX using the ZINC database targeting the dUMP-substrate binding space, followed by crystallographic studies.^51^ The study showed that the imidazo[4,5-*d*]pyridazine derivative 48 displayed the highest inhibitory activity towards Mtb-ThyX, thereby making it a promising scaffold in the design of novel antimycobacterial agents.

## 5. Imidazopyridazine moiety in approved and investigational drugs

**Fig. 15:**
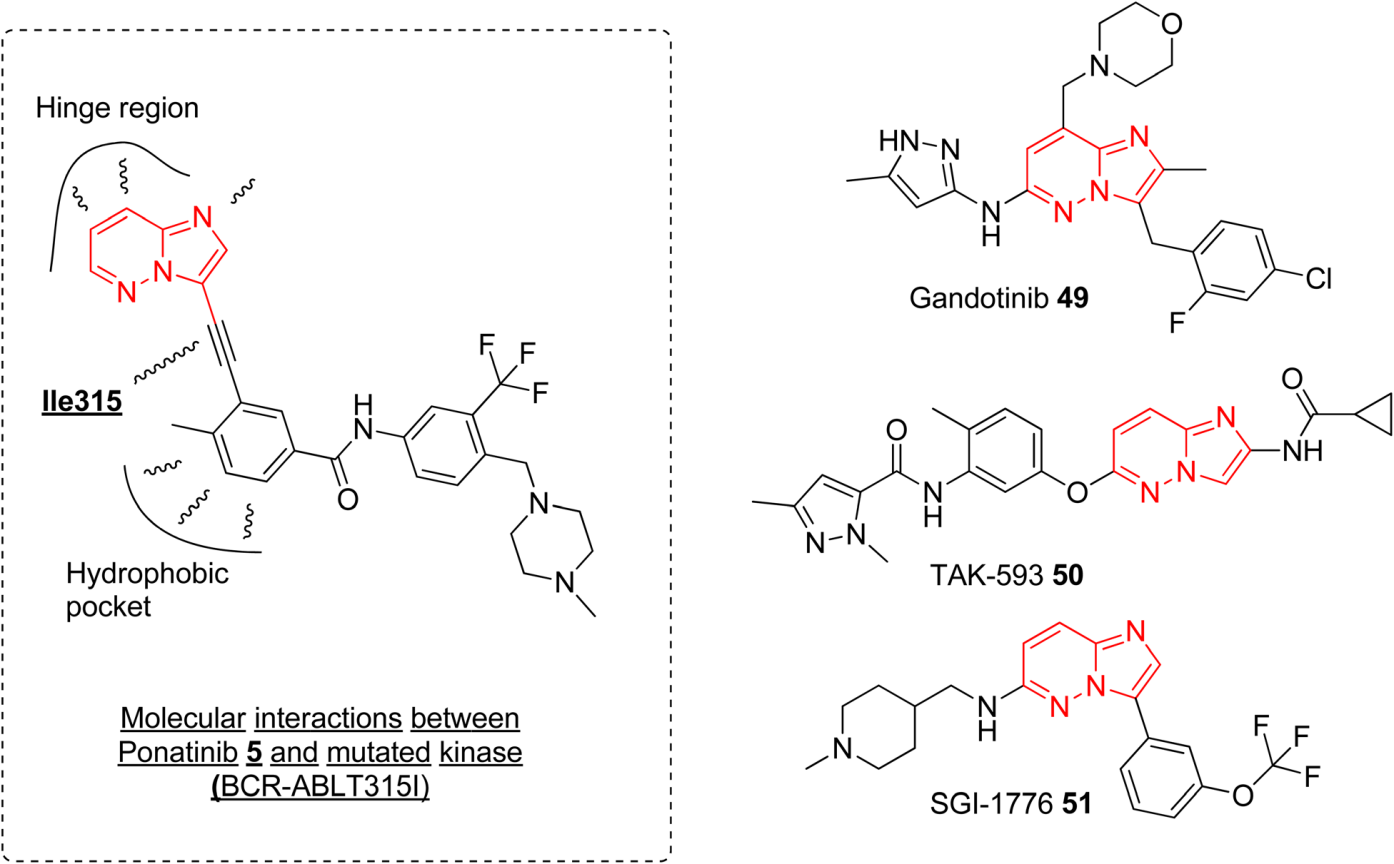
Imidazopyridazine moiety in approved and investigational drugs.

Currently, ponatinib 5 is the first and only imidazopyridazine derivative that has been approved by FDA, in 2012, as a drug for treating chronic myeloid leukemia (CML). It is indicated for use in treating adult patients with CML resistant to previous kinase inhibitors during various phases, *i.e.*, chronic, accelerated blast phases.^10,52^ The primary target of ponatinib is Bcr-Abl tyrosine kinase protein that stimulates and promotes the advancement of CML. In addition to targeting tyrosine kinase protein, ponatinib also inhibits other kinases (SRC, KIT, RET, TIE2, and FLT3) and receptors (VEGFR, PDGFR, FGFR, EPH).^53^ It is orally administered and approximately 64% of the dose undergoes phase I and phase II metabolism, primarily mediated by enzymes such as CYP3A4 and also by esterases and amidases, resulting in a half-life of 24 h.^54^ The discovery of ponatinib started with the research endeavor of Huang and co-workers working to tackle the resistance posed by the T315I mutation in kinase inhibitors.^55^ SAR studies of new BCR-ABL pan-inhibitors resulted in ponatinib that targets both native and mutated BCR-ABL kinase very effectively with IC_50_ value as low as 1.2 nM. Molecular docking studies revealed that the ethynyl group between the aromatic rings of 5 avoids steric clash with Il315 confirming more structural rigidity and less bulkiness than other established drug (imatinib). The binding modes of 5 were like imatinib primarily engaging in hydrophobic interactions within the hydrophobic pocket and forming additional favorable van der Waals interactions with Ile315 (Fig. 15). The enhanced inhibitory effect observed in BCR-ABLT315I-expressing Ba/F3 cells with ponatinib compared to imatinib was credited to the ethynyl spacer, which enables ponatinib to accommodate the Ile315 side chain. In addition to ponatinib, some of the imidazopyridazine derivatives were taken as investigational drug tested in various various phases of clinical trials. Clinical investigations of the imidazopyridazine derivatives that are available in public domain are discussed here.

Janus kinase (JaK) is a class of non-receptor tyrosine kinase and the dysregulation of JaK2 signaling due to mutation causes various myeloproliferative neoplasms.^56^ Gandotinib 49 is janus kinase 2 (JAK2) inhibitor that is being developed by Eli Lilly to treat various myeloproliferative neoplasms (MPNs). The phase I clinical trial was completed in 2018 and the safety, tolerability, and recommended dose were assessed for patients with JAK2V617F-positive myelofibrosis, essential thrombocythemia, or polycythemia vera.^57^ The study was conducted on 38 patients and the maximum-tolerated dose was 120 mg daily. The maximum plasma concentration was reached in *ca.* 4 h and the half-life was around 6 h. 29% of the myelofibrosis patients showed clinical improvement and spleen length reduction was observed in 20 out of 32 patients. Grade 1 severity of side effects such as diarrhea and nausea were observed. Overall, the drug demonstrated a pharmacological profile that supported further clinical development (https://clinicaltrials.gov/ identifier: NCT01134120). A phase 2 clinical study was conducted to assess the efficacy, safety, and pharmacokinetics of gandotinib in 138 MPNs patients with a daily administered dose of 120 mg. The studied Grade 3 or 4 treatment-emergent adverse events were observed in 2.2–11.6% of the patients and the highest overall response rate of 95% was seen in patients with JAK2 V617F-mutated polycythemia vera (https://clinicaltrials.gov/ identifier: NCT01594723). At the one-year mark, 44% of patients experienced a significant ≥50% improvement, which warrants further exploration in larger clinical trials.

Another imidazopyridazine that underwent clinical study is TAK-593 (50) that is a potent inhibitor of potent VEGFR and PDGFR classes of kinase receptors.^11^ A phase I clinical study was conducted to assess the safety/toxicity profile and determination of maximum tolerant dose in patients with advanced nonhematologic cancer. The results of the completed study were not published and this investigational drug 50 was not taken up for further clinical evaluation (https://clinicaltrials.gov/ identifier: NCT00773929). Similarly, another imidazopyridazine derivative SGI-1776 was developed as Pim-1 inhibitor with excellent potency in nanomolar concentration. It also demonstrated good anti proliferative effect by induction of apoptosis and autophagy.^58,59^ It was taken forward for clinical study and a dose escalation study was conducted on patients with relapsed leukemias. However, potential QTc prolongation led to the discontinuation of further clinical studies (https://clinicaltrials.gov/ identifierNCT01239108).

## 6. Analogs of imidazopyridazines

Imidazopyridazines represent fused heterocycles containing imidazole and pyridazine rings with untapped pharmacological potential. Imidazopyrimidines (purines) are immediate structural analogs of imidazopyridazines, both naturally occurring and found in numerous pharmaceutical drugs (Fig. 1).^60^ In addition, different analogs of imidazopyridazine with varying structural or functional similarities are known with imidazopyridazines being one of the least explored scaffolds. Some of these analogs are imidazopyridines,^61^ imidazopyrizines,^62^ pyrazolo[3,4-*d*]pyrimidines,^63^ pyrazolopyridazines,^64^ pyrido[2,3-*d*]pyrimidines,^65^ and related structures. The biological and pharmacological properties of these analogs differ significantly depending on their specific structure and the substitutions on the core moieties and have been reviewed thoroughly.^61–65^

## 7. Conclusions

Nitrogen-based heterocycles are core elements in the design and discovery of new drugs for treating a plethora of disorders and diseases. Imidazopyridazines are interesting heterocyclic scaffolds whose potential has not been fully investigated. They have primarily been established as kinase inhibitors in the design of anticancer and antimalarial drugs, which started with the FDA approval of ponatinib as a tyrosine kinase inhibitor for the treatment of chronic myeloid leukemia. In antimalarial drug design, imidazopyridazines have also demonstrated good prospects as kinase inhibitors with a few of them offering potential as drugs to treat malaria. In addition, imidazopyridazines have also been investigated as anticonvulsant, antiallergic, antihistamine, antiviral and antitubercular agents and in the treatment of psoriasis, thereby further establishing their pharmacological potential. Interestingly, three different structures of imidazopyridazines are possible and the imidazo[1,2-*b*]pyridazine variant is the most widely studied followed by imidazo[4,5-*d*]pyridazines. It is fascinating that imidazo[4,5-*d*]pyridazine and imidazo[4,5-*d*]pyridazine are more structurally closer to purine rings, a core moiety in many drugs. Research endeavors on imidazo[1,2-*b*]pyridazines provided encouraging results and few of them are in clinical development. Therefore, there appears to be substantial scope and opportunity to explore the potential of lesser-studied imidazo[4,5-*d*]pyridazines and imidazo[4,5-*d*]pyridazines as anticancer and antimalarial agents. Finally, imidazopyridazines have not been thoroughly studied for their potency towards resistant bacterial infections and there is a broad opportunity to do research in this direction based on the emergence of bacterial drug resistance.

## Conflicts of interest

There are no conflicts to declare.

## Supplementary Material
